# The Relationship Between Four Measures of Religiosity and Cross-National Variations in the Burden of Dementia

**DOI:** 10.7759/cureus.17034

**Published:** 2021-08-09

**Authors:** Ravi P Rajkumar

**Affiliations:** 1 Psychiatry, Jawaharlal Institute of Postgraduate Medical Education and Research, Pondicherry, IND

**Keywords:** dementia, alzheimer’s disease, religion, spirituality, depression, resilience, brain awareness, telomere length, social capital

## Abstract

Background

Several researchers have identified a possible protective effect of religiosity on the risk of dementia. Specific aspects of religiosity may be associated with this attenuation of risk, and it may be partially mediated through an effect on depressive symptoms or social support. However, this effect has only been demonstrated in selected cohorts to date.

Methods

This study was based on a cross-national analysis of associations. Correlations between World Health Organization estimates of the burden of dementia and four survey-derived measures of religiosity were examined across 101 countries, while controlling for estimates of late-life depression and social capital.

Results

Specific aspects of religiosity, such as attendance at religious services (Pearson’s r= -0.57), daily prayer (r* = -*0.58), and perception of religion as very important (r = -0.65), were associated with lower national levels of Alzheimer’s and other dementias (p< 0.01 for all correlations). This effect was partially mediated through an inverse relationship between religiosity and depression, but remained significant even after controlling for it and on multivariate analyses (β = -0.38 to -0.57, p< 0.01 for all measures). There was no evidence for a mediating effect of social capital.

Conclusions

Specific religious beliefs and practices may have a protective effect on dementia risk at the population level. These may involve group effects that require further study, such as reductions in depression in the elderly, or may involve beneficial effects on the stress response and cellular ageing in vulnerable individuals; however, the latter cannot be inferred with certainty from a group-level analysis. These results are consistent with earlier research and suggest a potential role for religious-based preventive strategies at the population level.

## Introduction

Dementia is a term used to refer to a group of generally irreversible conditions characterized by marked impairments in several domains of cognition. Alzheimer’s disease (AD), a neurodegenerative disorder with a distinctive pathophysiology characterized by the accumulation of amyloid neuritic plaques and neurofibrillary tangles, is the leading cause of dementia worldwide [1]. A significant proportion of the risk for dementia, and particularly for AD, is determined by genetic factors [2]. However, the development and subsequent progression of dementia is influenced by several environmental factors, some of which, such as diet, exposure to environmental pollutants, and comorbid conditions such as depression or diabetes mellitus, are amenable to modification [3,4]. Likewise, certain factors have been identified as potentially protective against the development of dementia, including specific dietary patterns, levels of specific nutrients such as folate, and involvement in physical or cognitive activity [5,6]. The protective effect of religious belief and practice against cognitive deterioration, and specifically against dementia, has attracted a certain amount of research interest in the past decade [7]. For example, affiliation with specific religious groups has been associated with a reduced risk of AD [8], while engagement in specific religious behaviors, such as daily prayer, has been associated with a reduced risk of cognitive decline at a later age [9]. Similarly, studies of patients with AD have found that religious activity is associated with better functioning in cognitive domains such as memory, language and constructional abilities, as well as with a slower rate of cognitive decline [10,11].

Two significant questions arise from an analysis of this research. First, what are the mechanisms that mediate the protective effect of religiosity on cognitive decline and dementia? Though no research has addressed this specific question to date, it has been observed that religious involvement in adults is associated with lower levels of depressive symptoms, which are positively correlated with telomere length [12]. This cellular process is of particular interest because shorter telomere length is significantly associated with an increase in the risk of certain types of dementia, such as AD [13]. Moreover, depression, particularly in late life, is a well-established risk factor for the development and progression of dementia [3,14,15]. Thus, the protective effect of religiosity on dementia could be mediated through its mitigating effect on depressive symptoms, as a relationship has been observed between the severity of these symptoms and the risk of AD [16]. Second, which are the aspects of religiosity that may exert a protective effect against cognitive decline in general and dementia in particular? This question is of significance because religiosity cannot be considered as a unitary construct: specific factors such as commitment to religious beliefs, the practice of prayer or meditation (“contemplation”), and engagement in altruistic behavior may have distinct effects on health. [17] Research in older adults has shown that religious attendance is negatively correlated with depressive symptoms, while private religious practice had no or even opposite effects on these symptoms, and the effects of religious contemplation on depression vary depending on whether an individual has a high or low genetic risk for this disorder [17-19]. Likewise, a study of elderly men from Israel found that religious education and observance was associated with a paradoxical increase in the risk of AD, in contrast with the majority of studies reporting a protective effect of religious practice against dementia [20]. Another important indirect mechanism that needs to be considered in such analyses is the relationship between religion and social support, particularly at the community level. Though religious and social capital cannot be directly equated with each other, there is evidence for a significant link between the two [21]. Though religiosity can be associated with a positive effect on health via increased social capital, a paradoxical effect can also be observed in some individuals and groups, with religious norms or practices being viewed as a constraint, leading to reductions in well-being [22].

Therefore, any analysis of the relationship between religiosity and AD should take into account both the different aspects of religious belief and practice, and the moderating effects of depression and social cohesion. The current study aimed to address these factors by examining the correlations between four different aspects of religiosity and the disease burden due to dementia across countries, while controlling for the effects of late-life depression and social capital.

## Materials and methods

Data sources

The current study was a cross-national association study. There are no large-scale estimates of the prevalence of dementia across nations. However, disability-adjusted life years (DALYs) for a given disorder provide a reasonable approximation to disease prevalence, when adjusted for population size and life expectancy, as DALYs are calculated based on either estimated incidence or estimated prevalence [23]. Information on DALYs for Alzheimer’s and other dementias, provided as an aggregate, were obtained from the World Health Organization’s Global Health Estimates for the year 2017, which provided estimated total DALYs for dementia for 183 countries and regions of the world. Raw values for DALYs were extracted from this source. As these values were computed for each country as a whole, they were divided by the estimated population for each country to provide adjusted DALYs for Alzheimer’s and other dementias (DALY-Dem), which were used as the independent variable in this study. Information on population sizes at the time the estimates were calculated was provided in the WHO dataset [24]. These DALYs were calculated based on the estimated prevalence of each particular disorder. Complete technical details of the calculation process are available in the WHO’s technical report on this data set [25].

To obtain information on various aspects of religiosity, data was obtained from the Pew Research Center’s 2018 publication, entitled “The Age Gap In Religion Around The World”, This report, based on surveys of individuals from a total of 105 countries and regions, obtained information on four aspects of religiosity:

1. religious affiliation;

2. attendance at religious services at least once a week, in those countries where weekly religious attendance is the norm in the majority religion (Christianity, Islam or Judaism);

3. daily participation in prayer;

4. respondents’ perception of religion as being “very important” in their lives.

All these responses were coded as dichotomous (yes/no) variables, and data for each country was provided as the percentage of positive responses. For example, among respondents in the United States of America, 77% reported affiliation to a particular religion, 36% reported weekly attendance at religious services, 55% reported praying at least once a day or more often, and 53% considered religion to be a very important aspect of their lives. In countries where weekly religious attendance is not the norm, such as India and Japan, the second of these variables were omitted [26]. This data set was selected because of its coverage of a large number of countries, and because it covered several distinct aspects of religious belief and practice, such as affiliation and prayer, which were identified as potentially important in earlier research. As this data was available for 101 of the 183 countries in the WHO data set, and as religiosity was the primary independent variable of interest, these 101 countries were selected for analysis in this study.

To analyze the possibility of a mediating effect of late-life depression on the link between religiosity and dementia, DALYs for depression for all adults aged 60 and above were obtained from the WHO Global Health Estimates [24] and adjusted for population size (DALY-Dep). This variable provides an indirect estimate of the prevalence of late-life depression [25]. Likewise, to assess the potential confounding effect of social cohesion, scores on the Social Capital pillar of the Legatum Prosperity Index were obtained from the World Bank’s database. This variable provides a composite measure of family and community networks, social cohesiveness, and level of trust in the government and other institutions. Scores on this index range from 0 to 100, with higher scores indicating greater social capital [27].

A complete list of all the countries included in this study, with data on measures of dementia, depression, religiosity and social capital, is included in Table 1.

**Table 1:** Raw data used for bivariate and multivariate analyses. DALY-DEM: population-adjusted DALY for Alzheimer’s and other dementias; DALY-DEP: population-adjusted DALY for depression in adults aged 60 and above; Rel-Affil: religious affiliation; Rel-Attend: weekly attendance at religious services; Rel-Pray: daily prayer; Rel-Important: religion considered “very important”; N/A: data not available for the respective country.

Country	Rel-Affil	Rel-Attend	Rel-Pray	Rel-Important	Life Expectancy	Social Capital	DALY-DEM	DALY-DEP
Afghanistan	100	61	96	92	65	N/A	19.5371	0.405319
Albania	99	7	15	15	79	46.53	68.5257	0.737851
Algeria	99	48	88	73	77	42.27	57.3391	0.653058
Argentina	89	20	40	43	77	51.97	25.9373	0.67883
Armenia	98	10	45	53	75	41.51	64.4103	0.946976
Australia	57	17	18	18	83	67.6	88.4513	0.866774
Austria	84	11	8	12	82	61.77	70.9282	0.726522
Azerbaijan	100	2	76	38	73	42.93	31.8203	0.670179
Bangladesh	100	54	57	80	73	44.19	21.5703	0.748953
Belarus	97	16	25	21	74	N/A	79.0284	1.25488
Belgium	62	6	11	11	82	58.47	124.754	0.953756
Bolivia	96	42	56	71	72	49.7	38.4598	0.782262
Brazil	92	45	61	72	76	52.93	37.1312	0.877154
Bulgaria	95	9	15	19	75	46.16	33.3193	1.16751
BurkinaFaso	100	N/A	N/A	93	62	48.9	16.1152	0.609112
Cameroon	98	70	82	90	59	48.87	21.9322	0.78796
Canada	67	20	25	27	82	66.23	98.5658	0.650306
Chad	97	77	83	86	54	43.07	18.3353	0.772152
Chile	84	19	39	41	80	51.15	39.5101	0.766578
China	13	1	1	3	77	41.55	83.1589	1.03326
Colombia	94	50	73	77	77	51.09	18.6425	0.646877
CostaRica	91	52	78	76	80	54.97	32.1958	0.800518
Croatia	93	24	41	42	78	45.61	63.8135	1.14022
Czechia	28	7	9	7	79	48.93	62.7006	1.06864
Dem Rep Congo	96	78	69	88	61	N/A	23.4234	0.634666
Denmark	70	3	10	9	81	64.49	106.015	0.80037
Djibouti	100	87	87	89	67	N/A	27.0938	0.843594
Dominican Rep	82	48	74	78	74	54.72	39.7449	0.761234
Ecuador	95	38	63	76	77	49.66	14.6951	0.722411
Egypt	100	62	72	72	72	42.73	42.0503	0.645085
ElSalvador	88	61	77	85	73	48.15	65.87	0.755188
Estonia	55	2	9	6	78	49.73	46.6112	1.22095
Ethiopia	100	82	65	98	67	44.49	18.509	0.754114
Finland	78	4	18	10	82	62.81	230.562	1.02238
France	72	12	10	11	83	53.71	113.492	1.02203
Georgia	100	17	38	51	74	42.04	101.016	0.956398
Germany	76	10	9	10	81	63.21	104.621	0.87386
Ghana	99	84	76	89	64	48.09	21.9859	0.687885
Greece	96	16	30	56	82	47.27	67.7143	0.955406
Guatemala	94	75	82	89	74	55.2	13.4222	0.661368
Guinea-Bissau	100	81	83	91	58	N/A	15.3805	0.909283
Honduras	90	64	78	90	75	48.41	28.9696	0.546374
Hungary	79	9	16	14	76	45.58	80.2587	1.1919
India	100	N/A	75	80	70	48.37	26.0625	0.918895
Indonesia	100	72	84	93	72	61.88	50.0402	0.362357
Iran	100	38	87	78	77	49.67	51.4841	0.837567
Iraq	100	42	87	82	71	N/A	23.8168	0.454869
Ireland	85	20	19	22	82	63.09	82.2126	0.794026
Israel	97	30	27	36	83	54.27	51.7549	0.734828
Italy	85	23	21	21	83	53.01	121.644	0.873735
Japan	44	N/A	33	10	84	46.98	94.5045	0.925204
Jordan	100	64	76	85	75	50.58	38.9594	0.274095
Kazakhstan	95	22	20	22	73	46.41	35.366	0.885384
Kenya	100	81	79	87	67	61.48	12.3624	0.627162
Kyrgyzstan	98	21	24	47	72	54.23	8.3949	0.657594
Latvia	79	6	18	11	75	47.81	51.7504	1.1527
Lebanon	100	35	51	57	79	43.64	107.533	0.724917
Liberia	100	79	80	90	64	47.7	16.1533	0.79646
Lithuania	94	9	15	16	76	44.09	47.2381	1.23342
Malaysia	99	45	61	77	76	56.76	50.0082	0.697277
Mali	100	79	81	94	59	47.54	20.2599	0.459382
Mexico	93	45	40	45	75	44.05	19.0905	0.640608
Moldova	98	15	49	42	72	44.18	33.867	1.15369
Morocco	100	55	80	91	77	39.76	59.2417	1.02361
Mozambique	87	84	68	87	61	48.21	19.7035	0.822104
Netherlands	51	12	20	20	82	62.07	135.879	0.91574
Nicaragua	93	55	75	88	74	52.55	45.4845	0.595798
Niger	99	88	87	86	62	N/A	14.8862	0.616876
Nigeria	100	89	95	88	55	51.52	25.6729	0.881544
Norway	57	7	18	19	83	65.06	106.061	0.804389
Pakistan	100	59	67	94	67	42.3	20.8512	0.677379
Panama	93	48	69	61	79	57.7	22.8752	0.681703
Paraguay	99	32	82	56	74	49.36	50.2763	0.626023
Peru	96	36	51	73	77	50.34	45.4304	0.522839
Philippines	100	53	82	91	71	59.57	11.7412	0.369085
Poland	93	42	29	30	78	48.36	33.1145	0.901472
Portugal	85	25	38	36	81	54.87	107.662	1.07379
Romania	99	24	45	50	75	47.58	40.5299	1.04536
Russia	85	7	18	16	73	45.37	70.1112	1.19845
Rwanda	99	80	62	90	69	47.39	17.2921	0.699888
Senegal	100	69	88	98	68	49.28	17.7764	0.576252
Serbia	96	7	27	34	76	N/A	57.3308	1.13403
Slovakia	75	23	31	23	77	48.28	77.9364	0.958891
South Africa	93	55	52	75	64	55.81	42.0005	0.812857
South Korea	54	29	32	16	83	N/A	58.2176	0.957509
Spain	70	15	23	22	83	57	133.051	0.843629
Sweden	58	6	11	10	83	61.31	140.86	1.02645
Switzerland	79	11	8	9	84	61.64	123.312	0.820514
Tajikistan	100	31	48	50	71	48.8	20.1714	0.333199
Tanzania	99	82	56	93	65	50.12	18.8612	0.668812
Tunisia	100	47	67	78	77	42.38	95.2244	0.906417
Turkey	99	44	60	68	78	46.82	105.761	0.742656
Uganda	100	82	66	86	63	53.8	13.7045	0.793234
Ukraine	93	17	30	23	72	42.49	85.1167	1.33946
United Kingdom	77	8	6	10	81	62.22	168.417	0.918095
United States	77	36	55	53	79	65.45	127.902	0.912895
Uruguay	63	14	29	29	78	57.57	76.387	0.769681
Uzbekistan	99	9	26	29	72	N/A	9.02312	0.582761
Venezuela	93	26	47	67	72	43.61	15.3241	0.755696
Vietnam	36	N/A	14	18	75	51.54	63.9787	0.574915
Zambia	99	86	78	91	64	47.98	13.7311	0.59566

Data analysis

In order to minimize the possible confounding effect of life expectancy on DALY estimates, particularly in low- and middle-income countries, DALY-Dem and DALY-Dep values were standardized in a linear manner for an ideal maximum life expectancy of 90 years. For this purpose, data on national life expectancies were obtained from the World Bank’s global database [28]. All study variables were tested for normality using the Shapiro-Wilk test; as they did not conform to a Gaussian distribution (p < 0.05 for all variables), they were transformed using a logarithmic transformation. As transformation using natural (base e) logarithms still yielded significant deviations from normality, the base 10 logarithm was used to transform each variable included in this study.

Bivariate analyses were carried out using Pearson’s correlation coefficient (r) to examine potential relationships between DALY-Dem and all four aspects of religiosity, as well as between these variables, DALY-Dep and social capital. All tests were two-tailed, with the threshold for significance set at p < 0.05 after applying Bonferroni’s correction for a 7 x 7 table. To identify potential multicollinearity between study variables, a threshold value of r ≥ 0.8 was used. In the event of multicollinearity between two or more variables, the variable showing the strongest individual correlation with DALY-Dem was included in the multivariate analysis. The strengths of observed correlations were graded according to standard guidelines as follows: poor (r < 0.3), fair (0.3 < r < 0.6), moderate (0.6 < r < 0.8) and very strong (r > 0.8) [29].

In order to test whether the relationship between dementia and religiosity was primarily mediated through variations in depression or social capital, a partial correlation analysis was undertaken between DALY-Dem and all four indices of religiosity, controlling for DALY-Dep and social capital, both individually and in combination. In these analyses, the significance level was set at p < 0.05 after applying Bonferroni’s correction for a 5 x 5 table.

Variables identified as significantly associated with DALY-Dem in bivariate analyses were included in a multivariate linear regression analysis, to identify the relative contributions of each variable to variations in DALY-Dem and the significance of each association. To assess for multicollinearity in this analysis, the variance inflation factor (VIF) was computed for each independent variable, and the data was re-analyzed after excluding any variable with a VIF greater than or equal to 4.

## Results

Data on a total of 101 countries were included in the final analysis. The results of unadjusted bivariate analyses are presented in Table 2. It was observed that in these unadjusted analyses, all four indices of religiosity were negatively correlated with DALY-Dem, indicating a possible direct or indirect protective effect. All these associations remained significant after corrections for multiple comparisons. The magnitude of these correlations ranged from “fair” to “moderate”. Among these four indices, the strongest negative correlation was observed for the perception of religion as very important (r = -0.65, p < 0.01) and the weakest was observed for religious affiliation (r = -0.43, p < 0.01). There was a modest positive correlation between DALY-Dep and DALY-Dem (r = 0.47, p < 0.01). DALY-Dep was significantly correlated with three indices of religiosity - religious service attendance, daily prayer and perception of religion as very important - but not with religious affiliation, and the strength of these associations was “fair”. It was also noted that there was significant multicollinearity between religious service attendance, daily prayer and perception of religion as very important (r = 0.84-0.92 for correlations between these variables); though religious affiliation was positively correlated with these variables (r = 0.59-0.70), this did not reach the threshold of concern for multicollinearity. Social capital showed a trend towards a positive correlation with DALY-Dem, but this was not significant after correction for multiple comparisons (r = 0.29, p = 0.235). There were no significant correlations observed between social capital and indices of religiosity.

**Table 2:** Bivariate correlations between country-wise DALYs for dementia, measures of religiosity, DALYs for depression in adults aged over 60, and social capital. DALY-DEM: disability-adjusted life years for dementia; DALY-DEP: disability-adjusted life years for depression in those aged 60 or above. *denotes a significant correlation at p < 0.05 after applying Bonferroni’s correction.

Variable	DALY-DEM	Religious affiliation	Religious service attendance	Daily prayer	Religion considered “very important”	DALY-DEP	Social capital
DALY-DEM	-	-0.43*	-0.57*	-0.58*	-0.65*	0.47*	0.29
Religious affiliation		-	0.59*	0.70*	0.70*	-0.27	-0.14
Religious service attendance			-	0.84*	0.89*	-0.49*	-0.18
Daily prayer				-	0.94*	-0.47*	-0.13
Religion considered “very important”					-	-0.52*	-0.25

The results of partial correlation analyses are presented in Table 3. Even after controlling for depression above the age of 60, significant negative correlations were observed between DALY-Dem and all four indices of religiosity, though the magnitude of all these correlations was reduced and remained in the “fair” range (r = -0.36 to -0.54). These associations remained significant after correction for multiple comparisons, and the pattern observed was similar to that seen in the uncorrected analyses, with the strongest correlation reported for perception of religion as very important, and the weakest for religious affiliation. A similar pattern emerged when controlling for social capital, and when controlling for both depression and social capital.

**Table 3:** Partial correlations between country-wise DALYs for dementia and measures of religiosity, conditioned on DALYs for depression in adults aged over 60 and social capital. DALY-DEM: disability-adjusted life years for dementia; SC: social capital. All correlations are partial Pearson’s correlation tests. *denotes a significant correlation at p < 0.05 after applying Bonferroni’s correction.

Variable	Religious affiliation	Religious service attendance	Daily prayer	Religion considered “very important”
DALY-DEM, conditioned on DALY-DEP	-0.36*	-0.44*	-0.46*	-0.54*
DALY-DEM, conditioned on SC	-0.39*	-0.58*	-0.54*	-0.62*
DALY-DEM, conditioned on both	-0.28*	-0.42*	-0.38*	-0.47*

In view of significant multicollinearity between religious service attendance, daily prayer and perception of religion as very important, the latter was selected for inclusion in multivariate analyses as it showed the largest magnitude of correlation with DALY-Dem in both direct and partial correlation analyses. For the purposes of multivariate linear regression analysis, DALY-Dem was selected as the dependent variable and DALY-Dep, religious affiliation and perception of religion as very important were entered as independent variables. Social capital was not entered as an independent variable in this analysis as it did not attain the threshold for significance in bivariate analyses. The results of this analysis are presented in Table 4. The final model explained approximately 43% of the variation in DALY-Dem (R^2^ = 0.45; adjusted R^2^ = 0.43). In this model, both DALY-Dep (β = 0.18, p = 0.048) and perception of religion as very important (β = -0.57, p < 0.001) were significantly associated with DALY-Dep, while the association with religious affiliation was not significant. Variance inflation factors ranged from 1.4 to 2.5 for each variable, ruling out significant multicollinearity between these variables.

**Table 4:** Multivariate linear regression analysis of variables associated with disability-adjusted life years for dementia. DALY-Dep: disability-adjusted life years for depression above the age of 60. *denotes significance at p < 0.05.

Variable	Regression coefficient (β)	Significance level (p)	Part correlation	Variance inflation factor (VIF)
DALY-Dep	0.18	0.048*	0.15	1.4
Religious affiliation	0.01	0.890	0.01	2.0
Perception of religion as very important	-0.57	<0.001*	-0.36	2.5

As a secondary measure, similar multivariate linear regression analyses were carried out including DALY-Dep and religious affiliation as independent variables, but using each of the other indices of religiosity (weekly religious attendance and daily prayer) in turn. Each of these models explained around 37% of the variance in DALY-Dem (adjusted R^2^ = 0.37 for both models). In the model including weekly religious attendance, DALY-Dep (β = 0.25, p = 0.008) and religious attendance (β = -0.38, p < 0.001), but not religious affiliation, were significantly associated with DALY-Dem. In the model including daily prayer, DALY-Dep (β = 0.25, p = 0.006) and daily prayer (β = -0.41, p = 0.001) were associated with DALY-Dem, while affiliation was not significantly associated with dementia. VIFs for all variables in these analyses were in the range of 1.3-2.4, indicating no significant concerns regarding multicollinearity.

## Discussion

The results of this preliminary analysis suggest that various aspects of religiosity may exert a protective effect against dementia, and may account for a modest proportion of the variations in the burden of dementia across countries. In general, this effect was stronger for measures of religious practice and belief, such as attendance at religious rituals, daily prayer and perception of the importance of religion, and somewhat weaker for affiliation with a given religion. These associations remained significant when controlling for a measure of late-life depression, as well as in multivariate analyses.

The associations between different aspects of religiosity and dementia observed in this study are consistent with prior research, which has identified a potentially protective effect of specific religious behaviors or practices on the onset and progression of dementia in general and AD in particular [9-11]. In other words, practices such as daily prayer or meditation, rather than membership status in a particular religious denomination, may have a greater and more significant protective effect. It was also observed that the largest potential protective effect against dementia in this dataset was observed for individual perceptions of religion as very important, indicating that the subjective aspects of religiosity are of equal if not greater importance than engagement in specific rituals. The high level of multicollinearity between variables measuring the aspects of religiosity did not permit a more specific examination of the relative contributions of each variable. Moreover, as the data analyzed was at the population level, it was not possible to capture inter-individual or intra-national variations in religiosity (for example, between economically developed or deprived regions, or between urban and rural areas) and their impact on dementia.

These results also suggest that religiosity is significantly and negatively correlated with depression at a population level. This suggests that countries or regions characterized by a higher level of religious practice may have lower levels of depression in the elderly, and this may in turn exert a positive influence on the subsequent risk of dementia at a group level. This finding is in line with the results of research in individual subjects [8,30]. However, given that the correlations between indices of religiosity and DALYs due to dementia remained significant in partial correlation and multivariate analyses, it is likely that other mechanisms may be involved in mediating this association. These may include behavioral factors, such as greater involvement in prosocial and altruistic behaviors related to religious belief, which has been shown to positively influence cognition in older adults [31]. Alternately, religious participation has been shown to exert a beneficial effect on diurnal variations in cortisol secretion [32]. Elevations in morning cortisol have been found to be associated with both mild cognitive impairment and AD, and it is possible that some aspects of religious belief or practice may attenuate such elevations and thus reduce the risk of cognitive deterioration, independent of their effects on depressive symptoms [33]. In this connection, it has also been observed that increased cortisol reactivity is associated with telomere shortening, which is a potential risk factor for some forms of dementia [13,34]. A further possibility that deserves consideration is that religiosity was associated with greater telomere length in individuals with a genetic risk of dementia, suggesting that religiosity may attenuate the risk of dementia associated with specific allelic variations [35]. On the basis of these studies, is plausible that protective effects on telomere length, or on related mechanisms of cellular ageing, may represent a “final common pathway” through which specific aspects of religious practice and behaviour could attenuate the risk of cognitive decline and dementia in the elderly [36]. These explanations should be regarded as speculative, as they are based on research in individuals, while the data in this study was derived on entire populations.

Given the difficulties in inferring causality from studies of association or correlation, indirect mechanisms mediating this association should also be considered. For example, specific genetic variants in certain populations may be associated a reduced risk of dementia independent of religious practice, or there may be an additive effect between the presence of “protective” genetic polymorphisms and specific religious activities, such as meditation [37]. The protective effects of religiosity may also be greater for specific religious groups or sub-groups, and may be critically influenced by factors such as gender and physical fitness [8]. Finally, engagement with religion may have indirect beneficial effects such as greater social support [38] or adherence to particular dietary patterns [39] both of which are independently associated with a reduced risk of cognitive impairment [40,41]. Data from the countries analyzed in this study suggests that the link between religiosity and dementia may not be mediated by social capital, though other indirect mechanisms at the population level still require investigation.

These results are subject to certain important limitations. First and foremost, this study was based on data from entire countries; therefore, while it is possible to extrapolate these findings to an individual level to a limited extent, this should be done cautiously to avoid the ecological fallacy. Second, data on the country-level burden of both dementia and depression was obtained from WHO estimates. As noted by the WHO researchers, these estimates are associated with a certain margin of error, particularly in low- and middle-income countries with lower levels of health infrastructure. Third, the WHO data provided aggregate DALYs for “Alzheimer’s and other dementias”; therefore, it is not possible to conclude whether the results of this study are valid for dementia in general, or for AD in particular. Fourth, information on religiosity was available only as aggregate responses or percentages, allowing for only a rough estimation of the different facets of religious belief and behavior, particularly in multi-cultural or multi-religious societies. Fifth, as religious affiliation was expressed as a simple dichotomous variable in the Pew Research dataset, it was not possible to assess the differential effects of specific religions, such as Islam, Hinduism, Buddhism, Judaism and Christianity, on dementia. This is significant because “protective” effects have been specifically reported for Christian and Buddhist religious practices, but an inverse relationship was noted in a sample of Jewish men [8,20,37]. Sixth, given the association-based design of this study, it is not possible to draw definite conclusions about a protective effect, or whether such an effect is direct or indirect. Seventh, because countries were chosen as the unit of analysis, it was not possible to examine the effects of individual-level variables, such as gender, on the associations reported here. Eighth, due to a lack of large-scale data, the analyses were not corrected for other factors, such as environmental pollution, which vary significantly across countries and are independently associated with the risk of dementia. Finally, the observed correlations were fair to moderate in size, indicating that religiosity is only one of a multitude of factors influencing the risk of dementia.

## Conclusions

Despite these limitations, these results are of significance as they corroborate the findings of research in individual subjects, and support the contention that certain aspects of religiosity may have specific protective effects against cognitive decline and dementia in the elderly at the population level. They also provide some confirmation of the hypothesis that a “protective” relationship of religiosity on dementia risk is partially mediated by the severity of depressive symptoms; however, no consistent evidence for a mediation through social capital was found in this study. Based on these results, it is possible that further studies of specific aspects of religiosity may lead to a better delineation of this putative protective effect, and whether it is direct or mediated through correlates of religiosity, such as healthy dietary practices. Analyzing the interaction of measures of religiosity with genetic and environmental risk factors at the individual level would enable a better understanding of the cellular mechanisms through which such protective effects are mediated, and whether they are directly due to religion or due to the incidental effects of an associated environmental, social or lifestyle factor. This may eventually lead to the development of religiously-informed preventive interventions at the group level, particularly in populations with a high genetic risk of dementia, as well as a greater understanding of how population-level factors may influence the onset or development of this group of disorders.
